# Causal analysis of self-sustaining processes in the logarithmic layer of wall-bounded turbulence

**DOI:** 10.1088/1742-6596/1001/1/012013

**Published:** 2018

**Authors:** H. J. Bae, M. P. Encinar, A. Lozano-Durán

**Affiliations:** 1 Center for Turbulence Research, Stanford University, CA 94305, USA; 2 School of Aeronautics, Universidad Politécnica de Madrid, 28040 Madrid, Spain

## Abstract

Despite the large amount of information provided by direct numerical simulations of turbulent flows, their underlying dynamics remain elusive even in the most simple and canonical configurations. Most common approaches to investigate the turbulence phenomena do not provide a clear causal inference between events, which is essential to determine the dynamics of self-sustaining processes. In the present work, we examine the causal interactions between streaks, rolls and mean shear in the logarithmic layer of a minimal turbulent channel flow. Causality between structures is assessed in a non-intrusive manner by transfer entropy, i.e., how much the uncertainty of one structure is reduced by knowing the past states of the others. We choose to represent streaks by the first Fourier modes of the streamwise velocity, while rolls are defined by the wall-normal and spanwise velocity modes. The results show that the process is mainly unidirectional rather than cyclic, and that the log-layer motions are sustained by extracting energy from the mean shear which controls the dynamics and time-scales. The well-known lift-up effect is also identified, but shown to be of secondary importance in the causal network between shear, streaks and rolls.

## 1. Introduction

Turbulence is an important nonlinear dynamical phenomenon and one of the most challenging problems in classical physics. However, it has not been systematically investigated from the point of view of causality and, to date, the only attempts are the works by [1, 2, 3]. Indeed, most standard methods in turbulence research (one-point statistics, correlations, Fourier analysis, mode decomposition, exact coherent structures, energy budgets, data interrogation, etc.) do not provide a clear causal inference between events. Understanding the causal relations in turbulent flows is a necessary requirement (although maybe not sufficient) in order to comprehend the physical behavior of the system in an intuitive manner. In this study, we investigate the causal interaction between different coherent structures in the logarithmic layer of fully developed wall-bounded turbulence.

Streamwise rolls and streaks are ubiquitous in wall-shear flows. They are considered to be the most representative coherent structures that are able to explain the dynamics of the flow as a whole. Since the experiment by [4] and the discovery of sublayer streaks and ejections by [5], among others, the roll-streak structure has attracted enormous interest within the fluid mechanics community. Their spatially and temporally varying structure is generally believed to play an important role in maintaining and carrying shear-driven turbulence [6, 7, 8, 9, 10, 11]. Most works have focused on the buffer layer where the streaks and rolls (or vortices) are essentially one-scale objects, which simplifies their analysis. Farther from the wall, streaks and rolls persist but their internal Reynolds numbers are higher, making their characterization and understanding a more challenging task.

Although it is widely agreed that in both the buffer and logarithmic layers the streaks and rolls are involved in a regeneration cycle, alternative mechanisms have been derived from different simplified scenarios. In the buffer layer, it has been hypothesized that streamwise vortices near the wall may collect the fluid from the inner region, where the flow is very slow, and organize it into streaks [12, 11]. Other alternatives suggest that the generation of streaks are due to the structure-forming properties of the linearized Navier–Stokes operator, independent of any organized vortices [13]. Conversely, the streaks are hypothesized to trigger the formation of vortices by losing their stability [10, 14]. For a review of self-sustained processes in the buffer layer see [15].

A similar but more disorganized scenario is believed to take place in the logarithmic layer [16, 17, 18]. The existence of a self-similar streak/roll structure consistent with the Townsend attached-eddy hypothesis [19] has been documented by [20, 21, 22], and [23] from direct numerical simulation data at moderate Reynolds numbers. However, the most interesting results are not the kinematic description of the structures in individual flow realizations, but rather the elucidation of how they relate to each other and how they evolve in time. A growing body of evidence indicates that the generation of these log-layer streaks has its origins in the linear lift-up effect [20, 24, 25, 26] in conjunction with the Orr’s mechanism [27, 11]. On the other hand, rolls are speculated to be the consequence of a sinuous secondary instability of the streaks that collapse through a rapid meander until breakdown [28, 29, 26, 30]. Other scenarios advocate for the formation of rolls via a stochastic structural instability [31].

The different mechanisms, each capable of leading to the observed turbulence structure, are rooted in theoretical or conceptual arguments. Whether the flow follows any or a combination of these mechanisms is in fact unclear. Most of the theories stem from linear stability theory, which has proved very successful in providing a theoretical framework to explain the lengths and time scales observed in the flow. However, an appropriate base flow for the linearization must be selected *a priori* depending on the flow state of interest, introducing some degree of arbitrariness. Moreover, quantitative results are known to be sensitive to the details of the base state [32]. Another criticism is that turbulence is a highly nonlinear phenomenon, and a full self-sustained cycle is not expect to be unraveled from a single set of linearized equations. For example, in turbulent channel flows, the classic linearization around the mean velocity profile does not account for the redistribution of energy from the streamwise velocity component to the cross-flow, which is the prevailing energy transfer on average [33]. In order to capture different energy transfer mechanisms, the base state for linearization should be selected accordingly. Moreover, optimal solutions should not be taken as representative of the actual flow and, if so, the time and length-scales for which linearization remains meaningful becomes a relevant issue, which is barely discussed in the literature. The causal analysis presented here aims to clarify what accounts for driving the fully nonlinear self-sustaining processes in the log-layer in a non-intrusive manner.

Causal inference is an important subject in many scientific disciplines. Given that what is usually known about events in question is in the form of time series, causal analysis of temporal signals is of particular importance. Frequently, causal inference is understood in terms of time-correlation between pairs of signals. However, it is well known that correlation lacks the directionality and asymmetry required to guarantee causation [34]. Another common method for causality assessment is the so-called Granger test [35], which is a statistical measure of the usefulness of one time series in forecasting another. However, this provides only a yes-or-no judgment, without the quantitative information that may be needed in many circumstances. In an attempt to remedy this deficiency, recent works have centered their attention to an information-theoretic measure, namely, transfer entropy [36]. This measure is notoriously challenging to evaluate, requiring long time series and a high associated computation cost [37]. However, recent advancements in entropy estimation from insufficient datasets [38, 39] have made transfer entropy a viable method to quantify causality.

The paper is organized as follows. The first section contains the methods subdivided in three parts (Section 2). First, we define transfer entropy as a tool to quantify causality. In the other two, we introduce the numerical experiment and the flow decomposition into mean flow, streaks, and rolls. The results are presented and discussed in Section 3 in terms of the characteristic time-scales for causality and the causal network between coherent structures. Finally, conclusions are offered in Section 4.

## 2. Methods

### 2.1. Quantitative causality analysis: transfer entropy

We use the framework provided by information theory to quantify the causality between a set of temporal signals by their associated transfer entropy [36]. Given a set of time-dependent random variables *x*_*k*_(*t*), *k* = 1,⋯*, n*, the transfer entropy from *x*_*j*_ to *x*_*i*_ is defined as

(1) $$T_{j\to i}(\Delta t)=H\left(x_{i}(t)|x_{1},\cdots ,j-1,j+1,\cdots ,n(t-\Delta t)\right)-H\left(x_{i}(t)|x_{1,\cdots ,n}(t-\Delta t)\right),$$

where *x*_1,⋯_,*n* = (*x*_1_,⋯*, x*_*n*_), and Δ*t* is the time-lag in the signal considered to evaluate causality at the current state. *H*(*x|y*) is the conditional Shannon entropy [40], that is, the uncertainty of a variable *x* given *y*, and is defined as

(2) H(x|y)=E[log(f(x,y))]−E[log(f(y))],

where *f*(·) is the probability density function, *f*(·|·) denotes the conditional probability density function, and *E*[·] signifies the expected value. In this form, the transfer entropy (or causality) from *x*_*j*_ to *x*_*i*_ can be interpreted as the decrease in uncertainty in *x*_*i*_ by knowing the past state of *x*_*j*_. We define the self-induced transfer entropy as *T*_*i*→*i*_, and the cross-induced transfer entropy as *T*_*j*→*i*_ for *j* ≠ *i*.

To guarantee statistical convergence, transfer entropy calculation requires data from long time series, especially when the dimension of the data set, *n*, is large. To alleviate the computational cost associated with computing *H*, the Shannon entropy is approximated using the Kozachenko-Leonenko estimator [38], which is known to provide reasonable improvements for high dimensional datasets from a relatively short time series [39].

Another key aspect for causality quantification is the normalization of the measurement. Time correlation, for example, guarantees a value between 0 and 1 when properly normalized, making the correlation coefficient relevant in terms of determining relative importance. In the same manner, causality must be normalized to allow for meaningful comparisons. We define the normalized transfer entropy [41] as

(3) $$NT_{j\to i}(\Delta t)=\frac{T_{j\to i}(\Delta t)-T_{j\to i}^{\text{Shuffled }}(\Delta t)}{T_{(1,\cdots ,n)\to i}(\Delta t)}.$$

The term $T_{j\to i}^{\text{Shuffled }}$ aims to remove spurious contributions due to statistical errors, and it is the transfer entropy computed from the random variables *x*_1_,⋯*, x*_*j−*1_,$x_{j}^{\text{Shuffled }}$,*x*_*j*+1_,⋯, *x*_*n*_ where $x_{j}^{\text{Shuffled }}$ is *x*_*j*_ randomly permuted in time in order to retain first order statistics but break any time-delayed causal links. Eq. (3) is used as the measure of causality for the remainder of the paper.

The causality measure from Eq. (3) is advantageous compared to the classic time-correlations. One desirable property is the asymmetry of the measurement, i.e., if a variable *x*_1_ is causal to *x*_2_, it does not imply that *x*_2_ is causal to *x*_1_. Moreover, as *NT*_2→1_(Δ*t*) is based on the probability density function of *x*_*k*_, it is invariant under shifting and rescaling of the signals. In addition, transfer entropy accounts only for direct causality, that is, if *x*_2_ is only caused by *x*_1_ and *x*_3_ is only caused by *x*_2_, there is no causality from *x*_1_ to *x*_3_ [41].

### 2.2. Numerical experiment

The data are obtained from direct numerical simulation of a plane turbulent channel flow with two periodic directions and no-slip condition at the wall [42]. In the following, the streamwise, wall-normal and spanwise directions are denoted by *x*, *y*, and *z*, respectively, and the corresponding velocity components by *u*, *υ*, and *w*. The friction Reynolds number of the simulation is Re_*τ*_ = *u*_*τ*_*δ/ν* = 934, where *u*_*τ*_ is the friction velocity, *δ* is the channel half-height, and *ν* is the kinematic viscosity.

The incompressible flow is integrated in the form of evolution equations for the wall-normal vorticity and the Laplacian of the wall-normal velocity [43]. The spatial discretization is dealiased Fourier in the two wall-parallel directions and Chebychev polynomials in *y*. Time stepping is third-order semi-implicit Runge-Kutta [44]. The number of grid points in the streamwise and spanwise directions are *N*_*x*_ = 128 and *N*_*z*_ = 128 such that the resolutions are Δ*x*^+^ = 11.5 and Δ*z*^+^ = 5.8, respectively, where the superscript + denotes wall units defined in terms of the friction velocity and the kinematic viscosity. The maximum wall-normal resolution is $\Delta y_{max}^{+}=7.7$. The simulation was run with *CFL* = 0.5 for 140*δ/u*_*τ*_ (after transients), and the velocity fields were stored every *~* 25 wall units to generate a time-resolved dataset.

The length, height and width of the computational domain are *L*_*x*_ = *πδ/*2, *L*_*y*_ = 2*δ* and *L*_*z*_ = *πδ/*4, respectively. These dimensions correspond to a minimal box simulation for the log-layer and are considered to be sufficient for isolating the relevant dynamical structures involved in the bursting process [16, 11]. Minimal simulation boxes have demonstrated their ability to reproduce statistics of full-size turbulence computed in much larger domains. [16] showed that turbulence remains “healthy” roughly below *y* ≈ 0.3*L*_*z*_, corresponding in our case to *y ≈* 0.25*δ*, that is the height chosen in the present study to analyze the causality transfer between rolls, streaks and mean shear.

**Figure 1.**
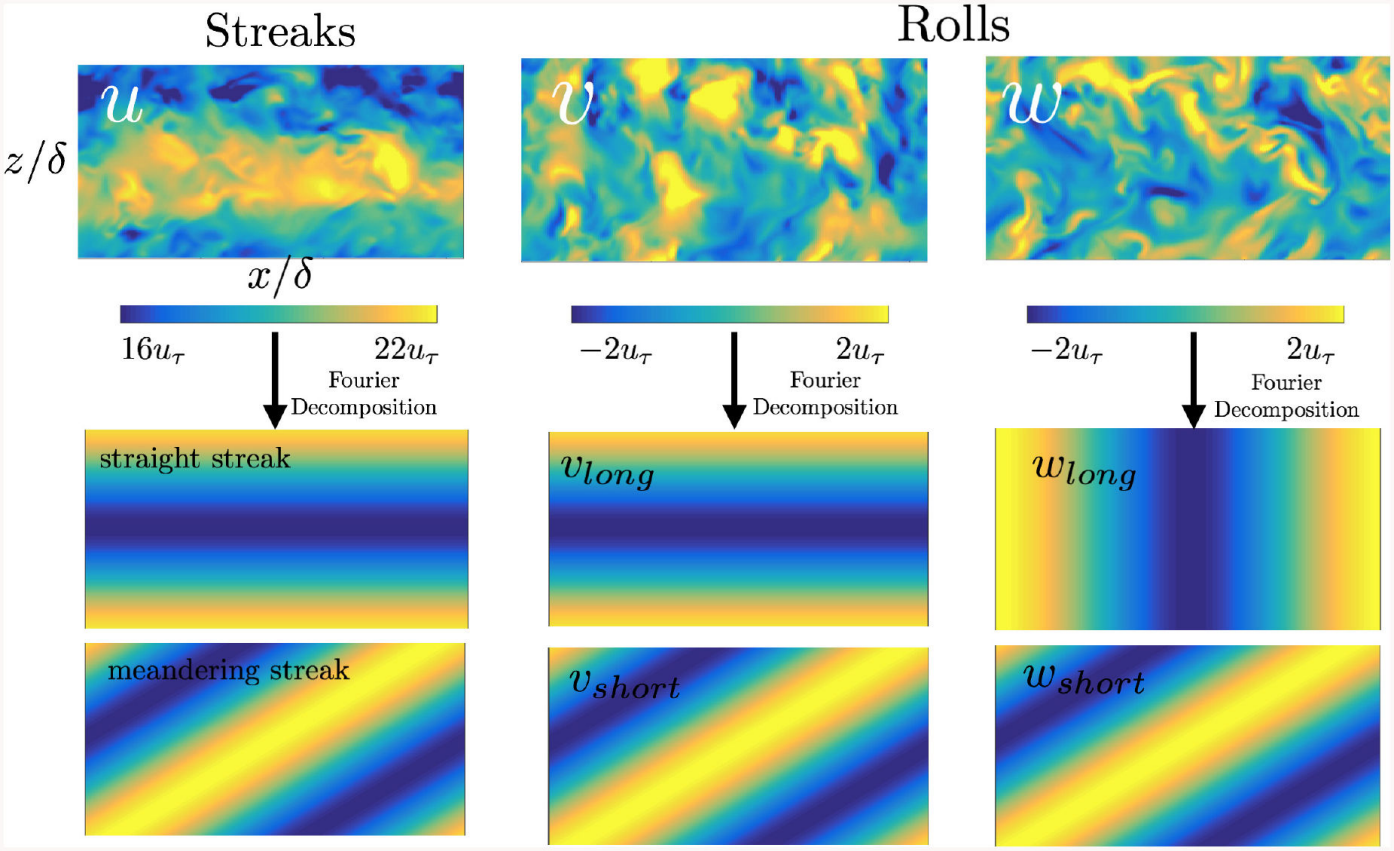
Snapshots of the instantaneous velocities *u, υ*, and *w* (top), and the representative Fourier modes of the streaks and rolls used for the causal analysis (bottom).

Figure 1 shows instantaneous snapshots of the three velocity components. The streaky elongated nature of *u* is clearly observed along the streamwise direction, as opposed to the shorter structure of *υ* and *w*. Figure 1 also highlights the fact that only a few large scale eddies are contained and isolated in this computational domain, enabling the causality analysis between individual objects.

### 2.3. Representation of streaks and rolls

We define ${\hat{u}}_{nm}(y,t)$ (respectively, ${\hat{v}}_{nm}$ and ${\hat{w}}_{nm}$) as the two-dimensional streamwise and spanwise Fourier transform of *u*(*x, y, z, t*) (respectively, *υ* and *w*) where $k_{x_{n}}=2\pi n/L_{x}$ and $k_{z_{m}}=2\pi m/L_{z}$ are the corresponding wavenumbers, with *n* = *−N*_*x*_*/*2*,…, N*_*x*_*/*2 − 1 and *m* = *−N*_*z*_*/*2*,…, N*_*z*_*/*2 − 1. The causal analysis is applied to snapshots of ${\hat{u}}_{nm}(y,t)$, ${\hat{v}}_{nm}(y,t)$ and ${\hat{w}}_{nm}(y,t)$ averaged in the range of wall-normal distances *y* ∈ [0.2*δ*, 0.3*δ*], consistent with the discussion in Section 2.2. Quantities averaged in the wall-normal direction are denoted by 〈·〉

**Figure 2.**
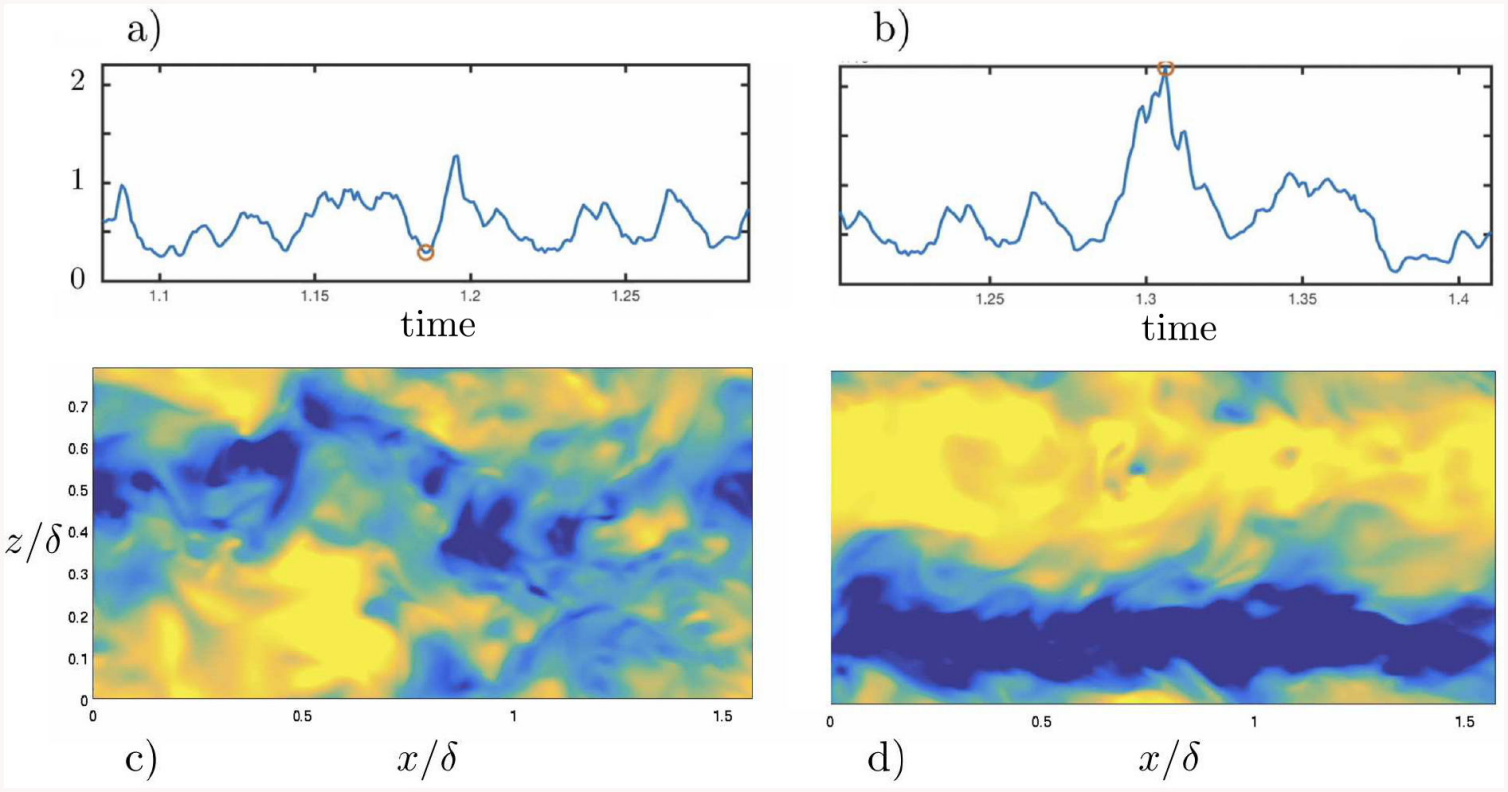
Two instances (red dot) in the time-series of the straight streak signal (a) and (b), and the corresponding streamwise velocity snapshots (c) and (d). Panels (a) and (c) feature a time of low intensity of the signal, whereas (b) and (d) correspond to the formation of a strong streamwise straight streak.

We focus the analysis on the shear-dominated motions carrying most of the turbulent kinetic energy. The mean time-varying velocity profile is defined as $\langle {\hat{u}}_{00}(y,t)\rangle$ and along the text we will occasionally refer to this component as *mean shear*. Streaks are characterized by the first modes of the streamwise velocity fluctuation, and are classified into two categories as shown in Figure 1. The straight streak is defined as the *L*_2_-norm of $\langle {\hat{u}}_{0(\pm 1)}(y,t)\rangle$. The meandering streak is represented by $\langle {\hat{u}}_{\left(\pm 1\right)\left(\pm 1\right)}\left(y,t\right)\rangle$ which are indicators of the streak breakdown. An example illustrating the two configurations of the streaks is shown in Figure 2. Rolls are defined as the cross-flow component of the velocity. Analogously to the streaks, $\hat{v}$ and $\hat{w}$ are divided into long and short motions (as shown in Figure 1). Long and short modes for $\hat{v}$ are (*n, m*) = (0,±1) and (*n, m*) = (*±*1*, ±*1), respectively. Whereas long and short modes for $\hat{w}$ are (*n, m*) = (±1, 0) and (*n, m*) = (*±*1*, ±*1). The final set of signals *x*_*k*_ for *k* = 1,⋯, 7, is composed of the streamwise velocity, the straight streak, the meandering streak, the long *υ*, the short *υ*, the long *w*, and the short *w*, respectively.

## 3. Results

### 3.1. Times-scales for causal interactions

The first goal is to establish the time horizon for causal influence between variables. This is achieved by computing the transfer entropy as a function of the time-lag, Δ*t*. The behavior of *NT*_*j→i*_(Δ*t*) may differ for each (*i, j*) pair, but for the sake of simplicity, we define two global measures: an average over all cross-induced transfer entropies ${\bar{NT}}_{j\to i}(\Delta t)\;(j\neq i)$ and self-induced transfer entropies ${\bar{NT}}_{i\to i}(\Delta t)$.

**Figure 3.**
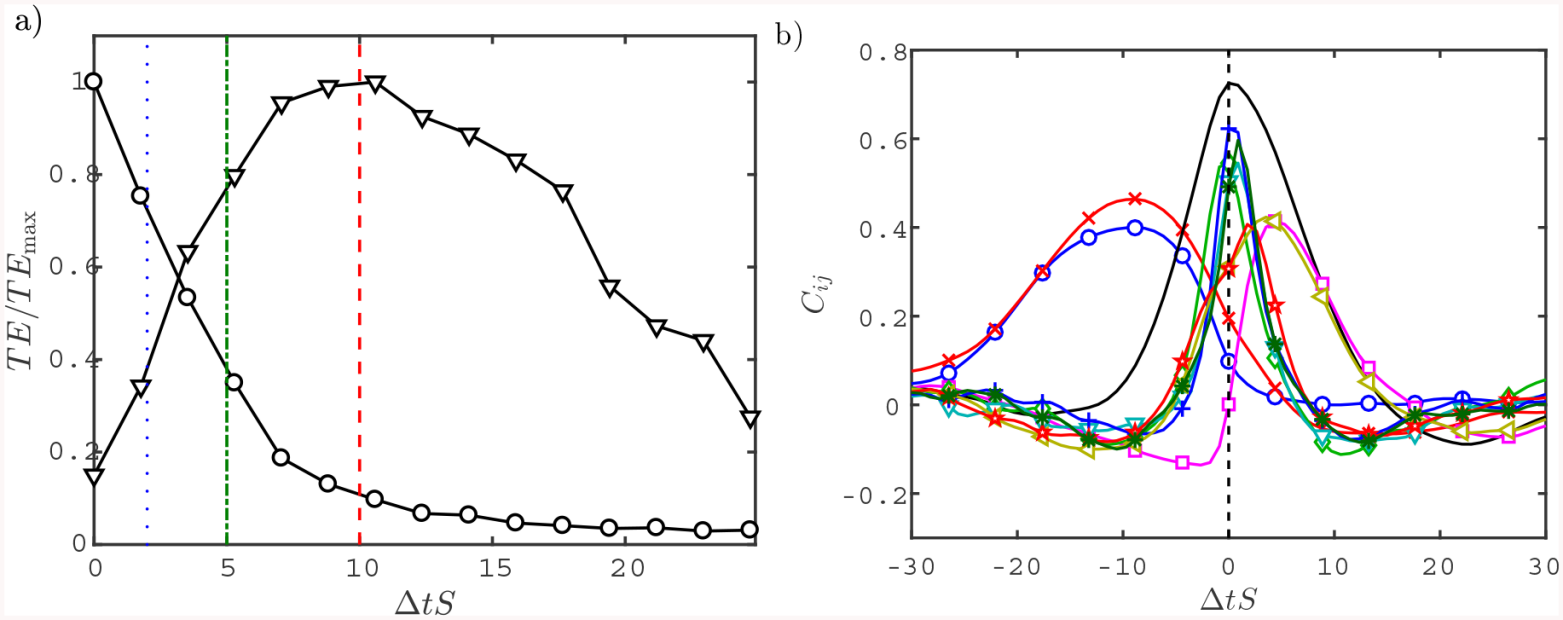
(a) Normalized self-(*○*) and cross-induced (∇) transfer entropy as a function of the time-lag. The blue dotted line, green dot-dash line, and red dash line are Δ*T* = 2*S*^−1^, 5*S*^− 1^ and 10*S*^−1^, respectively. (b) Time cross-correlations. Colors and symbols are: mean to straight streak (blue *○*); mean to *υ* long (red *×*); straight streak to *υ* long (black); straight streak to *w* long (magenta □); meandering streak to *υ* short (green ♢); meandering streak to *w* long (cyan ∇); meandering streak to *w* short (blue +); *υ* long to *w* long (gold ⊲); *υ* short to *w* long (red ⋆); *υ* short to *w* short (green *).

The results are shown in Figure 3(a), and times are normalized by the average shear, *S* =〈*∂u/∂y*〉. As expected, the maximum self-induced entropy peaks at Δ*t* = 0*S*^−1^, implying that the maximum reduction of uncertainty on a variable takes place by knowing the current state of the variable itself. More interestingly, the causality of a variable on itself has a characteristic time-span of *~* 5*S*^−1^ measured by the time ${\bar{NT}}_{i\to i}(\Delta t)$ has decayed by 50%. The average cross transfer entropy exhibits a different behavior, with a peak at Δ*t* = 10*S*^−1^ and a characteristic time-span of *~* 20*S*^−1^. The time scales discussed above are comparable to those in [45], who reported that the bursting time scale for shear flows is approximately universal and equal to 20*S*^−1^.

The previous analysis is analogous to the classic time-correlation of two variables that, for comparison purposes, is shown in Figure 3(b). Only cross correlations with a maximum value larger than 0.4 are included. The characteristic time scales of the time-correlations are of the order of 10*S*^−1^ and consistent with those from the transfer entropy analysis, meaning that both methods are valid to extract the most representative time scales of the flow. However, transfer entropy is specifically designed for building bi-directional causality maps among variables, whereas the time-correlation approach is limited by its symmetry for this purpose. The reader is referred to [46] for a more detailed analysis of the time-correlations in minimal channel flows

### 3.2. Causal network of the self-sustained streak-roll interaction

**Figure 4.**
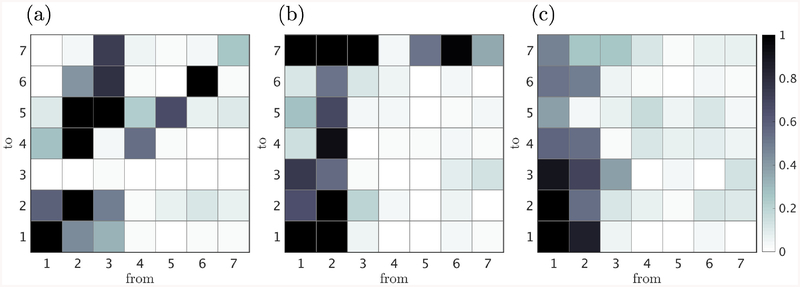
Causality maps for (a) Δ*t* = 2*S*^−1^, (b) 5*S*^−1^, and (c) 10*S*^−1^. Signal 1: time-varying mean velocity; 2: streamwise straight streak; 3: meandering streak; 4: long *υ*; 5: short *υ*; 6: long *w*; and 7: short *w*.

**Figure 5.**
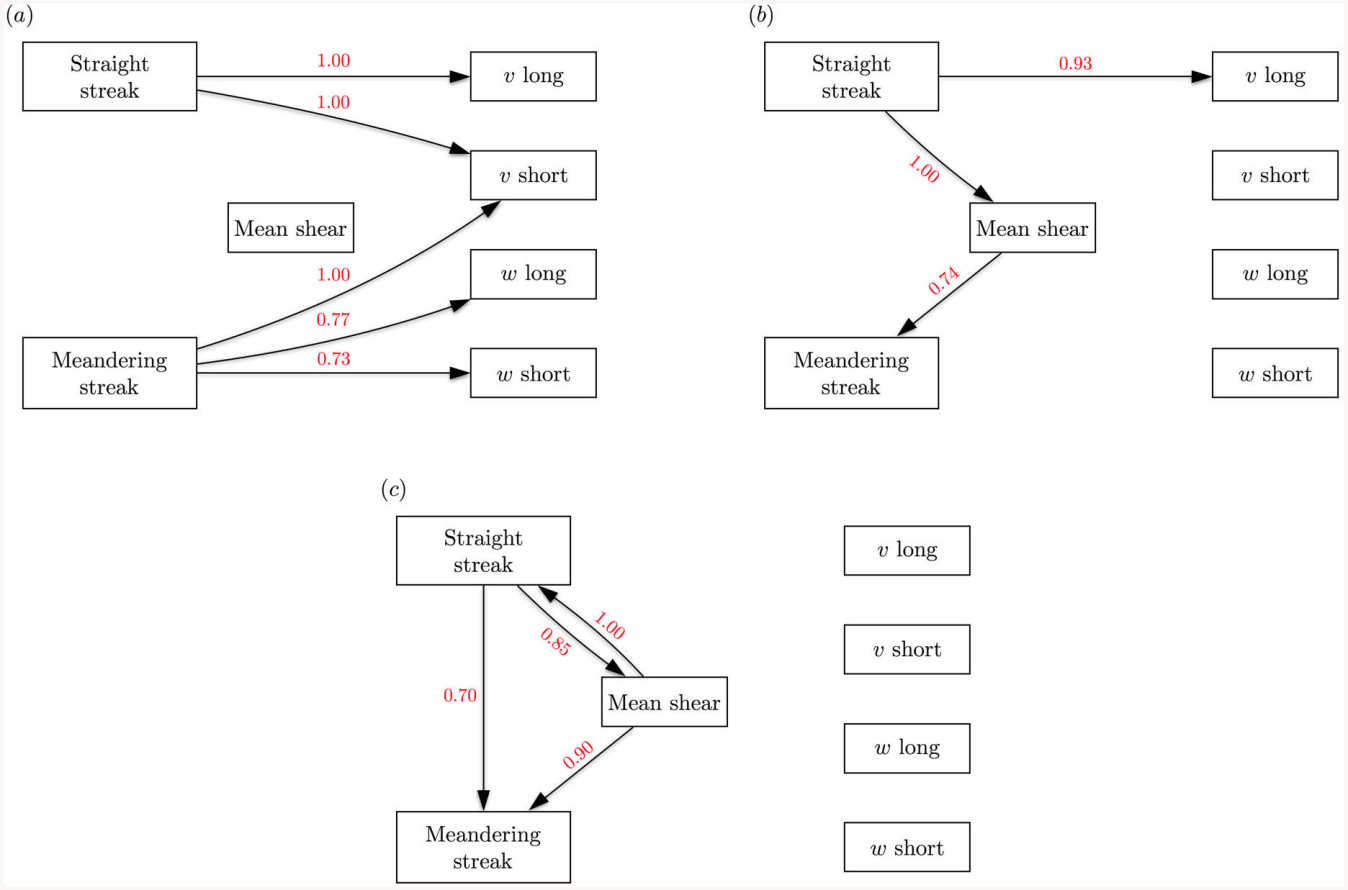
Causality diagrams for (a) Δ*t* = 2*S*^−1^, (b) 5*S*^−1^, and (c) 10*S*^−1^. Only connections with *NT >* 0.7 are included.

We examine the causal network (*NT*_*j→i*_,∀*j, i*) between variables at three different time scales, Δ*t* = 2*S*^−1^, 5*S*^−1^ and 10*S*^−1^. This choice aims to capture the transition in the causality network from a self-induced dominated to a cross-induced dominated scenario. Longer time-lags could be considered; however, the statistical errors become significant to draw meaningful conclusions and the extension to larger time scales will be relegated to future work. The full causality maps are shown in Figure 4 for the three time scales. As it could be anticipated given the complexity of turbulent flows, many causal relations are significant and there is no obvious dominant pattern. In order to attain a simplified physical interpretation of the results from Figure 4, the dominant cross-induced causal connectivities have been compiled in three causality diagrams (Figure 5). Dominant causalities are defined as those with *NT >* 0.7. In addition to this constraint and to avoid spurious solutions due to statistical errors, those causalities which did not remain above 0.7 when considering half of the time history were excluded.

The causality diagram for Δ*t* = 2*S*^−1^ is shown in Figure 5 (a). The diagram reveals that most of the information flows from the streamwise streaks to the cross flow components whereas the mean flow remains inactive. At Δ*t* = 5*S*^−1^ (Figure 5 b), the straight streaks are still the dominant element with a strong causal effect on the mean flow, which begins to influence the meandering streaks. Finally, at Δ*t* = 10*S*^−1^, the causality diagram is reverted and the causal effect flows from the mean flow to the streamwise streaks, while the effect of the streamwise streaks are lessened. For all three time scales considered, there is no causal influence from the cross flow to the other components. Many weaker causal influences (*NT <* 0.7) are omitted in the description above, the most significant one being the lift-up mechanism. This can be identified, for example, as the causal influence of the long *υ* to the straight streaks, that is also present and of the order ~ 0.3.

**Figure 6.**
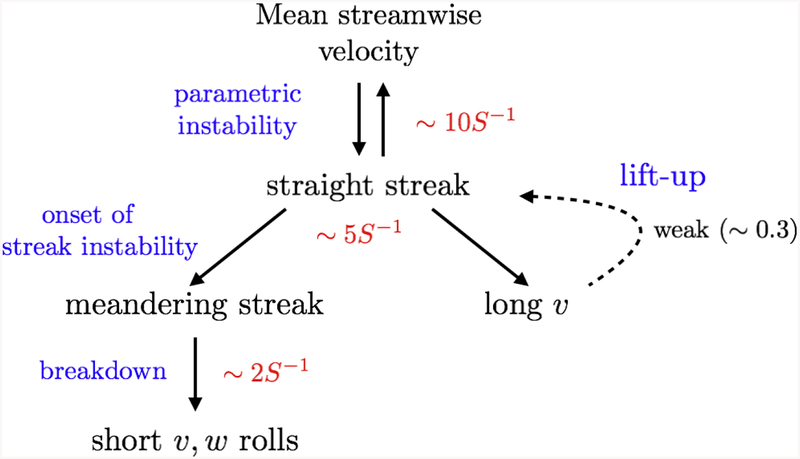
Summary of the most relevant causal relations for shear-dominated scales in the logarithmic layer. See text for details.

Finally, the self-sustaining process of the logarithmic layer is summarized in Figure 6 and interpreted in the context of different known mechanisms. The process is mostly unidirectional, emanating from the time-varying mean velocity profile, which generates straight streaks in a characteristic time scale of 10*S*^−1^. We refer to this process as a parametric instability. The effect of the straight streaks on the flow is twofold. First, it generates long velocity rolls which interact weakly (*NT <* 0.7) with the straight streaks through the lift-up mechanism. Secondly, straight streaks meander, marking the onset of its instability. Both processes take place at a time scale of 5*S*^−1^. The instability results in the breakdown of the streamwise streaks into short rolls, in a relatively fast process that spans along the time period of 2*S*^−1^.

It is important to remark that the causality discussed above is a measure based on the reduction of the statistical uncertainty of the variables, and it is not directly linked to energy transfer. Furthermore, the current method detects direct causality only if all the intermediate causal variables are accounted for. Otherwise, the causality may flow indirectly through other signals not taken into consideration. For example, the dynamic equation for ${\hat{v}}_{01}$ is

(4) $$\frac{\partial {\hat{v}}_{01}}{\partial t}=-\underset{n,m}{\sum }ik_{x_{n}}{\hat{v}}_{nm}{\hat{u}}_{\left(-n)(1-m\right)}-\underset{n,m}{\sum }\frac{\partial {\hat{v}}_{nm}}{\partial y}{\hat{v}}_{\left(-n)(1-m\right)}$$

(5) $$-\underset{n,m}{\sum }ik_{z_{m}}{\hat{v}}_{nm}{\hat{w}}_{\left(-n)(1-m\right)}+\nu \left(\frac{\partial^{2}{\hat{v}}_{01}}{\partial y^{2}}-k_{z_{1}}^{2}{\hat{v}}_{01}\right),$$

which does not directly depend on ${\hat{u}}_{0(\pm 1)}$ (similarly for ${\hat{v}}_{0(-1)}$). Hence, the causal connection from the straight streak to *υ* long shown in Figure 5 follows necessarily an indirect path.

## 4. Conclusions

Despite the extensive information provided by direct numerical simulation of turbulent flows, the causal relation between different flow structures has been overlooked in turbulence research. In the present work, we frame the causal analysis between turbulent signals from a transfer entropy perspective.

We have examined the causal structure between the streamwise rolls, streaks and mean shear in the logarithmic layer of a turbulent channel flow. The velocity components of the minimal channel of the log layer was decomposed in wall-parallel Fourier modes, resulting in a time series of the mean flow, two different configurations of streaks (straight and meandering), and rolls (short and long). Transfer entropy analysis was then conducted between the series, providing the causality flow between flow structures.

The detailed analysis of the causal network shows that the self-sustaining process is mainly unidirectional rather than cyclic, meaning that the logarithmic layer motions are sustained by the temporal change of the mean shear which controls the dynamics and time-scales. The causality flows then to the straight streaks which become unstable and break into smaller scale rolls. The well-known lift-up effect is present but shown to be weak from a causal point of view. The temporal causality horizon spans 20*S*^−1^ since the initialization of the process from the mean shear until the breakdown of the flow into smaller scale rolls.
